# Schizophrenia and dementia across the lifespan: epidemiological links, cognitive trajectories, and the pathophysiological interplay

**DOI:** 10.3389/fneur.2026.1779076

**Published:** 2026-04-07

**Authors:** Adam Bednorz, Dorota Religa

**Affiliations:** 1 John Paul II Geriatric Hospital, Katowice, Poland; 2 Institute of Psychology, Humanitas University, Sosnowiec, Poland; 3 Division of Clinical Geriatrics, Department of Neurobiology, Care Sciences and Society, Karolinska Institutet, Stockholm, Sweden; 4 Theme Inflammation and Aging, Karolinska University Hospital, Huddinge, Sweden

**Keywords:** Alzheimer’s disease, cognitive decline, dementia, frontotemporal dementia, schizophrenia

## Abstract

**Background:**

Schizophrenia is a severe psychiatric disorder characterized by persistent cognitive impairment across multiple domains and is increasingly associated with elevated risk of late-life dementia. However, the nature of this association and its underlying mechanisms remain unclear.

**Objective:**

This mini-review synthesizes current evidence on dementia risk in schizophrenia, focusing on epidemiology, cognitive trajectories, biological mechanisms, and differential relationships with Alzheimer’s disease (AD), vascular dementia (VaD), and frontotemporal dementia (FTD).

**Results:**

Epidemiological studies consistently indicate a two- to threefold increased risk of dementia among individuals with schizophrenia, although estimates vary due to diagnostic and ascertainment biases. Cognitive trajectories are heterogeneous: many patients remain cognitively stable over time, while subgroups demonstrate gradual or accelerated decline associated with negative symptoms, medical comorbidities, and social factors. Current evidence does not support a uniform progression toward Alzheimer-type neurodegeneration. Biomarker, neuropathological, and neuroimaging findings suggest distinct biological profiles, with reduced cognitive reserve, neurodevelopmental vulnerability, accelerated aging processes, and vascular and metabolic burden contributing to dementia risk. Genetic overlap between schizophrenia and AD appears modest, whereas partial clinical and molecular convergence is observed with FTD. Screening tools such as MMSE and MoCA may overestimate dementia prevalence due to longstanding baseline cognitive deficits. Sex differences, late-onset psychosis, and cardiometabolic comorbidities further modify risk trajectories.

**Conclusion:**

Dementia risk in schizophrenia likely reflects the interaction of lifelong neurodevelopmental vulnerability with aging-related and modifiable factors rather than a disorder-specific neurodegenerative pathway. Longitudinal biomarker-informed studies and tailored diagnostic frameworks are needed to improve differentiation between chronic cognitive impairment and true neurodegeneration.

## Introduction

Schizophrenia is a severe psychiatric disorder marked by chronic functional impairment and cognitive deficits constitute a core and enduring feature of the illness, affecting domains such as processing speed, attention, executive functions, memory, and social cognition, and they significantly contribute to disability across the lifespan (1–3). In older adults, these impairments become more pronounced and are increasingly recognized as a major driver of the elevated dementia risk observed in schizophrenia (4). This mini-review summarizes current evidence on dementia risk in schizophrenia encompassing Alzheimer’s disease (AD), vascular dementia (VaD), and frontotemporal dementia (FTD), with a focus on epidemiology, cognitive trajectories, and disorder-specific patterns across different dementia types. Literature searches were conducted primarily in PubMed, with a deliberate focus on longitudinal studies, meta-analyses, and review articles to capture long-term cognitive trajectories and dementia risk in schizophrenia (publications from 1994 to 2025). A more detailed description of the search strategy, including search terms, selection criteria, and study screening procedures, is provided in Supplementary material.

## Schizophrenia, adulthood, and the risk of dementia

Severe mental illnesses are associated with elevated dementia risk—highest in bipolar disorder, followed by schizophrenia and major depression (5, 6). Analysis of 86 million patients revealed age- and sex-specific comorbidity patterns in schizophrenia, including increased risks of delirium, dementia, and various somatic conditions in later life (7). A meta-analysis including over 12 million individuals showed that non-affective psychotic disorders more than double dementia risk (RR ≈ 2.5) (8). Large population-based and registry studies across multiple countries report a two- to threefold increased dementia risk in schizophrenia, particularly before age 65 and among individuals diagnosed in midlife (9–11). In a Medicare cohort, dementia prevalence rose from 28% at age 66 to 70% by age 80 in schizophrenia, compared with 1.3 and 11.3% in matched controls (12). A meta-analysis including more than 200,000 dementia cases in schizophrenia populations confirmed a significantly elevated risk of dementia, though with substantial heterogeneity (13). Propensity score–matched analyses further demonstrate elevated risk across dementia subtypes, including AD (aHR ≈ 2.1), with cardiometabolic, neurological, and substance-related comorbidities substantially amplifying vulnerability (9). Stronger associations have been observed in women and in older age groups, underscoring the growing public health relevance of schizophrenia–dementia comorbidity (14, 15). Overall, large registry and longitudinal studies consistently indicate that schizophrenia is associated with increased risk of subsequent dementia, although some cohorts of very old patients show attenuated or non-significant differences compared with age-matched controls (16–22). Furthermore, the elevated prevalence of dementia diagnoses in individuals with schizophrenia may partly reflect diagnostic misclassification and ascertainment bias rather than solely true neurodegenerative processes. Long-standing baseline cognitive deficits reduced cognitive reserve, cumulative antipsychotic exposure, and histories of institutionalization may lower the threshold for meeting clinical dementia criteria during normative aging, even in the absence of primary neurodegenerative pathology (2, 23, 24). Moreover, functional impairment—an essential component of dementia diagnosis—is frequently present in schizophrenia across the lifespan, which complicates the differentiation between chronic psychiatric-related disability and progressive neurodegeneration (24). The risk of dementia in schizophrenia patients may be partially overestimated due to diagnostic and ascertainment biases, though a genuine elevated risk likely remains. Stafford et al. (25) demonstrated that in very late-onset schizophrenia (LOS), the association was substantially attenuated (HR: 2.22) after accounting for potential misdiagnosis and ascertainment bias, compared to the unadjusted estimate (HR: 4.22). Moraiti and Porfyri (26) noted that studies have significant limitations including small sample sizes and selection of chronic older patients, Lyketsos et al. (27) emphasized that dementia development in schizophrenia is “not inevitable.” However, the risk of dementia in patients with schizophrenia is significantly higher compared to those without serious mental illness, as evidenced by a large retrospective cohort study involving over 8 million participants (28).

Emerging evidence suggests that antipsychotic exposure may be associated with increased dementia risk in schizophrenia, with one study reporting a 92% higher risk in individuals under 65 (HR = 1.92) (29), and others linking specific agents (olanzapine, risperidone, clozapine) to elevated AD risk, potentially via impaired microglial amyloid-β clearance (30); however, postmortem data have not confirmed increased Alzheimer-type neuropathology in treated patients (31). Treatment-resistant schizophrenia has been associated with selective working memory deficits and reduced hippocampal volume rather than global impairment (32). Importantly, dementia risk estimates in schizophrenia may be confounded by healthcare access and diagnostic disparities, as individuals with schizophrenia spectrum disorders are less likely to receive prior mild cognitive impairment (MCI) diagnoses or anti-dementia treatment and may experience delayed recognition of dementia (33–35). Accordingly, while epidemiological data robustly support an association between schizophrenia and dementia, they do not consistently indicate a disorder-specific causal link with AD, highlighting the need for longitudinal, biomarker-informed studies and refined diagnostic frameworks. Selected studies are presented in Table 1.

**Table 1:** Selected studies of dementia risk and cognitive decline in schizophrenia.

Study (year)	Sample size/cohort	Follow-up	Findings	Key conclusion
Hedges et al. (12)	Medicare registry; age 66–80	Cross-sectional registry	Dementia prevalence 28% → 70% vs. 1.3% → 11.3% in controls	Dramatically elevated dementia risk in schizophrenia
Ribe et al. (10)	Danish registry (>2.8 M; ≥50 years)	Up to 18 years	IRR ≈ 2.1 for dementia; strongest effect <65 years	Schizophrenia doubles dementia risk, especially in earlier-onset cases
Jonas et al. (42)	428 psychosis patients	30 years	~16-point IQ decline; deterioration began ~14y before onset	Supports dual neurodevelopmental + neurodegenerative model
Hendrie et al. (17)	31,588 older adults (1,635 with schizophrenia; mean age 70)	10 years	Dementia prevalence 64.5% vs. 32.1% in non-schizophrenia group	Markedly higher dementia prevalence and mortality in schizophrenia
Mentzel et al. (20)	168,780 individuals ≥65y (2,103 with schizophrenia; New Zealand)	7 years (registry)	Dementia 23% vs. 25% in controls	No significant increase in dementia prevalence in very old schizophrenia cohort
Teipel et al. (22)	1,686,759 dementia cases; 3,373,518 controls (Germany)	Mean 6.6 years	Schizophrenia associated with OR >2.5 for dementia	Among strongest clinical predictors of dementia diagnosis
Almeida et al. (16)	37,770 men aged 65–85 (dementia-free at baseline)	Up to 17.7 years	Sub-HR 2.67 (95% CI 2.30–3.09) for psychotic disorders	Nearly threefold increased dementia risk
Kershenbaum et al. (18)	28,340 adults ≥65 years in UK psychiatry services	6 years	Standardized dementia incidence rate 2.9 (1.5–4.7) for schizophrenia	Dementia incidence elevated vs. general population
Stevenson-Hoare et al. (21)	~1 M Welsh EHR + 228,937 UK Biobank participants	Longitudinal EHR	HR 2.87 (Wales) and 4.46 (UKB) for schizophrenia	Strong association between schizophrenia and later dementia
Kørner et al. (19)	12,616 late-onset + 7,712 very-late-onset schizophrenia (Denmark)	3–4.6 years	RR 2.21–3.47 vs. controls	2–3 × higher dementia risk in late/very-late schizophrenia

EHR, electronic health records; HR, hazard ratio; IRR, incidence rate ratio; IQ, intelligence quotient; M, million; RR, relative risk.

## Cognitive trajectories in schizophrenia

Despite the increased risk of dementia, cognitive decline in schizophrenia is highly heterogeneous. Cross-sectional meta-analyses consistently show large, generalized cognitive deficits in older individuals with schizophrenia, whereas longitudinal studies and meta-analyses largely support relative short- to medium-term stability (1–5 years), even in chronically ill and functionally impaired patients (36–40). Longitudinal research identifies distinct cognitive trajectories, including stable, gradually declining, and more rapidly deteriorating courses. Approximately half of patients remain cognitively stable, while smaller subgroups show modest or accelerated decline, particularly those with severe negative symptoms, recurrent psychotic episodes, treatment resistance, or institutionalization (2, 41–43). Fluctuations across cognitive domains may occur in relation to relapse–remission patterns. A systematic review of 23 longitudinal studies found mixed evidence for late-life cognitive decline. Of the three studies applying DSM/ICD dementia criteria, two reported increased dementia prevalence. Among studies assessing specific cognitive domains, results were heterogeneous, and many did not control key risk factors such as education, vascular burden, or apolipoprotein E (APOE) genotype (44). Furthermore, systematic reviews further support the existence of multiple cognitive phenotypes within schizophrenia spectrum disorders, including cognitively preserved, intermediate, and globally impaired subgroups (45). When decline occurs, it is typically modest and domain-specific, with fluid abilities more vulnerable than crystallized functions such as verbal knowledge, which often remain stable or improve. However, long-term studies exceeding 10 years remain limited and yield inconsistent findings (46).

Overall, evidence slightly favors the presence of late cognitive decline, but it remains unclear whether this reflects schizophrenia’s pathophysiology or superimposed risk factors.

## Cognitive screening in schizophrenia: validity considerations

Cognitive impairment is a core feature of schizophrenia, consistently demonstrated across multiple studies using screening tools such as the Mini-Mental State Examination (MMSE) (47) and the Montreal Cognitive Assessment (MoCA) (48). Patients with schizophrenia typically score in the range of 17.91–22.7 on the MoCA, indicating mild to moderate impairment. For instance, Amdur et al. (49) reported a mean of 22.7 ± 4.3 in 112 stable patients, while Rademeyer et al. (50) found 22.53 ± 3.91 in 30 outpatients, and Doğu et al. (51) reported 17.91 ± 3.83 in 135 patients. Across approximately 631 participants, Dedovic et al. (52) observed that 83.7% scored below 26 (53). These results contrast with population norms (27.4 ± 2.2), confirming that cognitive deficits are pervasive in schizophrenia (48, 49, 50, 51–53).

A meta-regression of 56 studies (*n* = 5,588) found that MMSE scores in schizophrenia declined by ~1 point every 4 years—five times faster than in the general population—primarily driven by institutionalized patients, whereas community-dwelling patients remained relatively stable (54). In 272 partially remitted outpatients, scores were 2–3 points below population norms across all ages, but gaps did not widen with age; performance was associated with education and race, with difficulties most common in memory, attention, and construction tasks (55). Middle-aged and older outpatients (*n* = 161) scored significantly lower than controls, with 23% scoring ≤24 on the MMSE. Impairments were most prominent in delayed recall and attention, and poorer scores were associated with lower education, structured living, unmarried status, and severe negative symptoms (56).

While MMSE identifies clinically relevant deficits, it may not capture subtle cognitive impairments that MoCA can detect. In small clinical samples (*n* = 30), MoCA demonstrated moderate sensitivity (41.7%) and specificity (66.7%) relative to MMSE (57). In a larger outpatient cohort (*n* = 64), MoCA effectively detected both mild and severe cognitive impairments (AUC ≈ 0.81–0.82), also explaining additional variance in functional outcomes (58). MoCA performance is also correlated with education, illness severity, and negative symptoms, and predicted length of hospitalization (59). Long-term follow-up studies following first-episode psychosis further confirmed MoCA’s concurrent validity with the MATRICS Consensus Cognitive Battery (MCCB), suggesting a < 25 cut-off for impairment in chronic psychotic populations (60).

We were unable to identify data reporting MoCA or specific MMSE scores, nor longitudinal changes in these measures, in patients with a prior diagnosis of schizophrenia who were subsequently diagnosed with dementia. In 73 hospitalized older patients with schizophrenia, 66% were classified as “demented” using the MMSE, indicating a high prevalence of cognitive impairment (61). In screening assessments, the usefulness of the Clock Drawing Test (CDT) has also been highlighted (62, 63), as well as the INECO Frontal Screening (IFS) (64).

Taken together, these findings emphasize that baseline cognitive deficits in schizophrenia can confound the interpretation of MMSE and MoCA scores, potentially leading to over- or underestimation of dementia risk. Therefore, clinicians should consider the limitations of these screening tools, as well as individual patient factors such as education, illness severity, and living environment, when assessing cognitive decline in schizophrenia.

## Sex differences and late-onset schizophrenia

Evidence consistently demonstrates sex differences in schizophrenia in age at onset, premorbid functioning, symptom profiles, and substance use. Men typically show earlier onset, poorer premorbid functioning, greater negative symptom severity, lower affective symptoms, and higher rates of alcohol or substance misuse, whereas findings on broader psychopathology, neurocognition, social cognition, and personal resources remain inconsistent (65). These sex- and age-dependent differences extend to dementia risk. In general schizophrenia populations, men show modestly elevated risk (incidence rate ratio 2.38; 95% CI 2.13–2.66) and higher dementia-related mortality (relative risk 5.19 vs. 2.40 for females) (10, 66). However, women with LOS (after age 40) exhibit the highest dementia risk, being 3–4 times more likely to develop dementia than earlier-onset cases, with age-specific patterns showing the greatest relative risk under 65 and peak absolute risk after 65 (10, 26). In women, psychotic symptoms fluctuate with hormonal changes: exacerbations occur during low-estrogen phases of the menstrual cycle, postpartum, or following estrogen-lowering treatments, and menopause is associated with increased hallucinations, delusions, reduced cognitive functions, and decreased antipsychotic efficacy (67, 68). Perimenopause represents a sensitive neurobiological period during which declining estrogen signaling disrupts cerebral bioenergetic regulation, resulting in a hypometabolic brain state that may heighten vulnerability to neurodegenerative processes later in life (69, 70).

Late-onset schizophrenia (LOS; onset >45 years) appears to retain a genetic vulnerability similar to earlier-onset forms, while very-late-onset schizophrenia-like psychosis (VLOSLP; onset >60 years) is increasingly conceptualized as a heterogeneous and possibly multifactorial condition (71, 72). VLOSLP has been associated with elevated mortality—largely due to physical comorbidities and accidents—and with a higher subsequent risk of dementia compared to non-psychotic controls (73, 74). VLOSLP is more common in women and is characterized predominantly by positive symptoms, lower genetic loading, and higher mortality largely linked to physical comorbidity and accidents (2). Some longitudinal data suggest that a subset of late-onset cases later develop dementia, most commonly AD, supporting the hypothesis that in certain individuals VLOSLP may represent a prodromal stage of neurodegeneration (25, 75, 76). However, this interpretation remains debated. When age is controlled for, very-late-onset psychosis patients do not differ cognitively from earlier-onset groups, despite exhibiting more pronounced positive symptoms (77), and dementia rates are not higher than in aged early-onset schizophrenia populations (74). Cognitively, LOS is most consistently characterized by executive and visuospatial impairments, with some evidence of decline emerging around age 65, although existing data are limited by small and frequently institutionalized samples (23). Moreover, late-life schizophrenia presents a cognitive profile distinct from both AD and late-life depression, marked by greater impairment in learning than recall (78). Together, these findings suggest that while late-onset psychosis may, in some cases, signal evolving neurodegeneration, it does not uniformly represent a primary dementing process and should not be automatically equated with it.

Compared with MCI, schizophrenia patients may show relatively preserved recall and naming but similar deficits in attention, verbal fluency, and visuospatial functioning, further complicating differential diagnosis (79). Collectively, these findings suggest that cognitive decline in schizophrenia reflects heterogeneous trajectories shaped by illness timing, baseline cognitive reserve, and aging-related processes, rather than a uniform progression toward neurodegeneration.

Although the mechanisms underlying the elevated dementia prevalence in schizophrenia remain unclear, proposed explanations include a high burden of medical comorbidities, intrinsic cognitive and brain dysfunction, partially shared etiological pathways with dementia, and the impact of antipsychotic treatment and adverse health behaviors (13). The high prevalence of somatic comorbidities [e.g., metabolic syndrome in 39% of patients >50 years further contributes to accelerated cognitive aging and dementia risk (80)]. Schizophrenia is marked by high rates of cardiovascular and metabolic comorbidities, social isolation, smoking, and physical inactivity, all known dementia risk factors (81–84). A systematic review and meta-analysis of 27 studies (*n* = 10,174) found that metabolic syndrome, diabetes, and hypertension are significantly associated with greater global cognitive impairment in schizophrenia, particularly affecting executive functions, memory, and attention, whereas obesity and insulin resistance showed weaker or nonsignificant effects (85). Consistently, comorbid metabolic syndrome and diabetes have been linked to more severe cognitive deficits (86). Shared mechanisms may involve gut–brain axis dysregulation, while the high prevalence of obesity in schizophrenia likely reflects multifactorial influences, including antipsychotic treatment, lifestyle, and genetic and psychosocial factors (87).

Multiple studies consistently demonstrate that men with schizophrenia experience significantly higher cardiovascular mortality than women, with cardiovascular disease representing the leading cause of death and associated with markedly elevated standardized mortality ratios (88, 89). This excess male mortality introduces the possibility of survival bias in sex-specific dementia estimates. As highlighted in broader epidemiological research, higher premature mortality among men may artificially inflate the observed prevalence of dementia in women, as fewer men survive to the ages at which dementia typically manifests (90). Sex-related differences in selective survival may therefore partially account for the reported higher incidence of dementia among women (91). Copeli et al. (92) acknowledge that survival bias cannot be excluded in older adults with schizophrenia, but empirical quantification of its impact remains lacking. Within schizophrenia populations, cognitive impairment severity significantly impacts outcomes, with severely impaired patients experiencing more hospitalizations due to relapse and lower quality of life compared to those with mild impairment (93).

## Schizophrenia, Alzheimer’s disease, and shared vulnerability: genetic and familial perspectives

In a large cohort of 486,297 UK Biobank participants, individuals with bipolar disorder (BD) and major depressive disorder (MDD) showed significantly increased AD risk (HR = 2.37 and 1.63, respectively), with schizophrenia demonstrating a borderline association, collectively suggesting that mental disorders may act as independent risk factors for AD through partially distinct pathogenic mechanisms (94). Genetic studies indicate a modest but positive association between schizophrenia and AD, alongside shared links of both conditions with lower general cognitive ability (95). A Mendelian randomization analysis based on large genome-wide association study (GWAS) demonstrated that genetic liability to schizophrenia is associated with an increased risk of all-cause dementia, AD, and VaD (96).

Twin studies consistently estimate substantially higher heritability for AD (71%) than GWAS, suggesting that a large proportion of genetic risk remains unexplained by currently identified common variants (97). Schizophrenia is a highly heritable disorder, although estimates vary substantially depending on study design and methodology. Large twin and population-based studies consistently report high heritability, typically ranging from ~70% to over 80%, including estimates of 79% in a nationwide twin cohort using methods accounting for censoring (98), 83% in a Finnish twin sample (99), 82–85% in the Maudsley Twin Register (100), ~80% in comprehensive review (3), and 0.67 (95% CI 0.64–0.71) in a Danish population cohort of over 2.6 million individuals (101). Genome-wide association studies similarly support substantial genetic liability, despite evidence for “missing heritability” not captured by common variants (102). In contrast, family-based designs yield lower estimates, with heritability of 31% in nuclear families and 44% in extended families (103), and analyses based on raw concordance data suggest more modest corrected estimates (~23%), highlighting the influence of shared environment and methodological assumptions (104). Meta-analytic evidence further supports substantial genetic contribution to schizophrenia liability and its overlap with heritable cognitive traits across large samples (>800,000 individuals) (105). Family and population studies robustly demonstrate high heritability of psychotic disorders, with risk increasing sharply with genetic proximity to affected relatives (106–108). Studies examining family history further show that familial loading for psychosis is associated with slightly earlier illness onset and greater severity of negative symptoms, with sex-dependent interactions supporting a diathesis–stress model rather than a deterministic genetic mechanism (65, 109).

Genomic analyses indicate no direct genetic correlation between schizophrenia and AD, but shared associations with social and environmental factors—such as loneliness, socioeconomic disadvantage, polygenic risk, and family psychiatric history—interact to modestly shape individual schizophrenia risk trajectories (110, 111). The finding that schizophrenia polygenic risk is inversely associated with psychosis in AD further argues against shared etiologic pathways (112). Within this framework, observed associations between schizophrenia, delusional disorder, LOS, and dementia may reflect a continuum of psychosis-related vulnerability rather than a direct inherited pathway specifically predisposing to AD (95). However, studies examined outcomes in offspring of parents with schizophrenia or cognitive features of schizophrenia, but none assessed dementia as an outcome (113). Existing studies focus on the risk of psychiatric disorders in children of parents with severe mental illness—showing a 32% probability of developing such disorders by adulthood (114). However, to our knowledge, we could not identify studies specifically examining dementia risk in offspring with schizophrenia in relation to family history. Environmental stressors may shape epigenetic patterns associated with schizophrenia risk; youth at familial high risk exhibit differences in epigenetic age, indicating altered biological aging trajectories, although the precise mechanisms underlying stress-related methylation changes remain unclear (115). Together, these findings suggest that any shared genetic vulnerability between schizophrenia and dementia is more likely mediated through indirect pathways and contextual factors than through overlapping, disorder-specific genetic architecture.

Sex-stratified genome-wide and transcriptome-wide analyses demonstrate marked sex differences in AD genetic architecture, including female-specific loci and pathways, indicating that susceptibility to neurodegeneration is partially sex-dependent (116, 117). In both autosomal dominant and sporadic AD, genetic risk factors—most notably APOE-ε4—interact with sex to shape cognitive trajectories and neuropathological burden, with stronger associations observed in women, particularly in later life (118–121). APOE-ε4 exhibits strong sex-dependent effects, with women—particularly in mid-to-late older adulthood—showing greater vulnerability to AD–related neurodegeneration than men, including increased tau accumulation, more pronounced hippocampal and temporal cortical atrophy, and greater cognitive and neuropsychiatric burden (118, 121, 122). Notably, even in cognitively normal individuals, female APOE-ε4 carriers demonstrate stronger default mode network disruption, hypometabolism, and cortical thinning, indicating a female-specific susceptibility detectable at preclinical stages of AD (123–125). The available evidence does not support a consistent association between APOE-ε4 and schizophrenia risk independent of AD pathology. Several case–control studies found no significant differences in APOE-ε4 allele frequency between patients and controls (126–128), and a meta-analysis concluded that APOE-ε4 is at most modestly associated with schizophrenia in Caucasian populations, without playing a major etiological role (129). Although one earlier study reported a higher APOE-ε4 frequency in schizophrenia (130), these findings have not been consistently replicated. Conversely, some data suggest that APOE-ε4 may even be less prevalent in specific subgroups, such as individuals with later age at onset (>31 years) (131). However, emerging longitudinal evidence suggests that APOE-ε4 may influence the clinical course of schizophrenia. In a 20-year follow-up of 116 individuals from first hospitalization, ε4 carriage was associated with age-related worsening of psychotic symptoms, particularly hallucinations and delusions (132). Although ε4 frequency does not consistently differ between patients and controls, it has been linked to symptom severity, poorer outcomes, suggesting a role in shaping positive symptom expression. However, findings remain inconsistent across populations, including conflicting results regarding age at onset (133). Furthermore, a meta-analysis of 29 case–control studies demonstrated a significant moderating effect of age, with the APOE-ε4–schizophrenia association becoming more apparent in older individuals (132).

A meta-analysis by González-Castro et al. (134), including 28 association studies (4,703 controls; 3,452 patients), found no overall association between APOE variants and schizophrenia. Notably, a protective effect of the ε3 allele emerged in Asian populations, whereas no associations were observed in Caucasian samples, underscoring ethnic variability and possible sex-dependent effects. Beyond genetic association, APOE may be mechanistically relevant. The ε4 allele has been linked to myelin breakdown and accelerated cognitive decline in AD, and myelin abnormalities are also reported in schizophrenia. Supporting this overlap, post-mortem studies demonstrate increased APOE expression in the dorsolateral prefrontal cortex—particularly Brodmann areas 9 and 46—regions implicated in executive dysfunction in schizophrenia (133). Although global gene expression patterns differ between AD and schizophrenia, both disorders exhibit overlapping molecular dysregulation in the superior temporal gyrus (BA22), likely involving autophagy pathways, suggesting a partially shared biological substrate (135). Genetic evidence further supports convergence in psychotic symptom risk: higher schizophrenia polygenic risk scores are associated with increased likelihood of delusions in AD, pointing to shared liability for psychosis across the two conditions (136). Inflammatory mechanisms may also overlap, as triggering receptor expressed on myeloid cells 2 (TREM2) expression in leukocytes is elevated in both AD and schizophrenia, despite no corresponding increase in TYRO protein tyrosine kinase binding protein (TYROBP). differences or direct schizophrenia-associated genetic effects, highlighting its potential as a cross-disorder biomarker (137). Taken together, current evidence indicates that APOE-ε4 is unlikely to represent a robust shared genetic risk factor for schizophrenia but may act as an age-dependent modifier of symptom progression and late-life cognitive vulnerability, potentially reflecting partial overlap with neurodegenerative mechanisms.

In schizophrenia, well-established sex differences are observed in age at onset, premorbid functioning, symptom profiles, and substance use, while findings in neurocognition and broader psychopathology remain inconsistent (65). Hormonal fluctuations across the female lifespan appear to influence symptom expression, with periods of reduced estrogen associated with greater psychotic severity and cognitive vulnerability (67, 68). Differential associations between dementia severity and molecular markers—such as opposing correlations of DEK proto-oncogene protein (DEK) expression in the anterior cingulate cortex in men versus women—suggest that neurodegenerative pathways may diverge by sex (138).

## Schizophrenia and Alzheimer’s disease: neuroimaging and biomarkers perspectives

Earlier neuropathological studies based on relatively small autopsy cohorts consistently failed to demonstrate an increased prevalence or severity of Alzheimer-type pathology—including amyloid β deposition and neurofibrillary tangles—in patients with schizophrenia, even among those with clinical dementia, suggesting that cognitive deficits in “pure” schizophrenia arise from biological mechanisms distinct from classical AD neurodegeneration, although these conclusions should be interpreted cautiously given the limited sample sizes (139–146).

Biomarker and neuropathological studies indicate that cognitive decline in schizophrenia cannot be simply equated with AD. Cerebrospinal fluid (CSF) profiles differ from the classical AD pattern: although reduced amyloid-β42 (Aβ42) levels are observed, tau concentrations are typically normal or even reduced, contrasting with the elevated tau characteristic of AD (147, 148). Peripheral biomarker data similarly suggest a distinct pattern, with lower serum total tau and p-Tau levels compared to controls (149). Proteomic analyses demonstrate a broad reduction across multiple Aβ isoforms rather than the selective Aβ42 decrease seen in AD, and amyloid-related markers appear associated with global cognition and treatment exposure (150).

Neuropathological findings are heterogeneous. While dementia severity in schizophrenia has been linked to neuritic plaques and hippocampal tangles—particularly among APOE-ε4 carriers, supporting a reduced cerebral reserve hypothesis (151)—only a minority of elderly patients meet full neuropathological criteria for AD (7.6% in one cohort), and many show either mixed pathologies or no definitive neurodegenerative substrate (152, 153). Structural imaging studies further demonstrate overlapping white matter microstructural abnormalities in schizophrenia and AD, associated with episodic memory and executive dysfunction, but these findings do not establish a uniform neurodegenerative process (154–156). Both schizophrenia and AD are associated with reduced hippocampal and amygdala volumes, although patterns of association vary by age of onset and negative symptom severity (157). Reduced gray matter volumes have been observed in the cingulate and orbitofrontal cortices, without clear correlations with cognitive or psychiatric symptom intensity (158). In older schizophrenia patients, comorbid dementia was associated with greater cognitive impairment and reduced hippocampal, amygdala, and thalamic volumes (159). In schizophrenia, hippocampal decline appears steeper than in healthy aging and correlates with cognitive and functional impairment, yet such reductions often emerge early and independently of dementia, limiting their specificity for AD and complicating differential diagnosis in later life (24, 160). Furthermore, neuroimaging studies reveal both overlapping and distinct brain network alterations in schizophrenia and AD. Resting-state functional connectivity analyses in 162 AD/MCI patients, 181 schizophrenia patients, and 315 controls showed shared and disorder-specific network abnormalities, driven mainly by default mode, visual, and subcortical networks (161). Structural MRI analyses similarly achieved 81% accuracy in differentiating the disorders and identified subcortical volumes—particularly the putamen—as key markers for classifying late-onset psychosis (162). Both conditions exhibited reduced thalamic nuclei volumes, with the right medial dorsal nucleus in schizophrenia associated with disorganized thought and auditory hallucinations (163). Altered fronto-parieto-limbic activation during emotional processing correlated with behavioural social dysfunction in both disorders, suggesting a shared neural substrate for social impairment independent of diagnosis (164). Additionally, lower plasma brain-derived neurotrophic factor (BDNF) levels have been associated with poorer cognitive performance, with reduced BDNF correlating with lower MMSE scores in chronic schizophrenia (165).

The discrepancy between increased dementia diagnoses and the absence of consistent neuropathological findings may reflect several mechanisms. These include neurobiological processes intrinsic to schizophrenia that are not detectable using standard postmortem markers, as well as an amplified impact of normal ageing due to reduced cognitive reserve. Additional risk factors—such as substance use, socioeconomic adversity, and long-term antipsychotic treatment—may impair cognition without producing macroscopic neuropathological changes (24).

Memory impairment—especially in delayed recall—remains a key overlapping feature in both conditions (166), although specific neuropsychological measures such as verbal memory and abstract reasoning may aid diagnostic differentiation (167). Within schizophrenia, cognitive heterogeneity is clinically meaningful. Better executive functions, working memory, and premorbid functioning have been associated with long-term remission (168), while nonremitted inpatients show greater impairments in processing speed, attention, and working memory (169).

Overall, these findings indicate that schizophrenia-associated dementia risk reflects a complex interplay of modest genetic overlap with AD, familial and sex-modulated vulnerability, environmental exposures, and neurobiological changes, rather than a straightforward convergence with classical Alzheimer-type pathology. Stratified analytic approaches considering sex, family history, and age at onset are required to disentangle inherited susceptibility from illness- and context-related effects.

## Schizophrenia and vascular dementia

Vascular dementia affects approximately 2.4–30% of patients with schizophrenia, with significant variation across studies. Park et al. (170) specifically found a 2.4% prevalence in a nationwide South Korean study, while Moraiti and Porfyri (26) reported a higher range of 30% in psychotic patients. In a large propensity score–matched cohort (6,040 patients with schizophrenia; 24,160 controls), schizophrenia was associated with a significantly increased risk of all-cause dementia (aHR = 1.80), particularly AD (aHR = 2.10) and VaD (aHR = 1.67), with cardiometabolic, neurological, and substance-related comorbidities further amplifying risk, while both first- and second-generation antipsychotics were paradoxically associated with a lower incidence of dementia (9). A large Taiwanese cohort study showed that schizophrenia is associated with a significantly increased risk of both AD and VaD (171). The relationship appears complex, with greatest dementia risk observed in patients with shorter duration of psychotic symptoms (≤5 years), while chronic patients (10 + years) show lower risk (26). Vascular cognitive impairment was more frequently diagnosed in LOS (172).

Genetic variants such as methylenetetrahydrofolate reductase (MTHFR) T and catechol-O-methyltransferase (COMT) Val alleles are linked to impaired folate/homocysteine metabolism, endothelial dysfunction, and poorer executive functions in schizophrenia, supporting a genetic contribution to vascular and cognitive deficits (173). Furthermore, genes that increase schizophrenia risk appear to be associated with higher stroke risk, though evidence is still emerging. Rødevand et al. (174) found extensive genetic overlap between schizophrenia and cardiovascular disease risk factors using genome-wide association studies, identifying shared loci across multiple pathways including blood pressure, lipids, diabetes type 2, and coronary artery disease. More specifically, Ibrahim et al. (175) identified the hsa-miR-146a C > G gene variant as associated with both schizophrenia and acute ischemic stroke in chronic schizophrenic patients, operating through neuro-inflammatory and oxidative stress pathways. However, the number of specific shared genetic variants identified remains limited, and most molecular evidence is recent. The genetic overlap appears complex, with mixed effect directions across different risk factors.

Dysfunction of the neurovascular unit—comprising microvessels, glial cells, pericytes, and neurons—may represent a shared mechanism linking schizophrenia with cardiometabolic conditions such as type 2 diabetes and metabolic syndrome. Neurovascular endotheliopathy and blood–brain barrier disruption may promote neuroinflammation and oxidative stress, contributing to cognitive and behavioral impairment (173). Additional mechanisms may involve dysregulated calcium (Ca^2+^) and cyclic adenosine monophosphate (cAMP) signaling pathways signaling affecting neurotransmission and neuronal survival (176), as well as increased vulnerability to oxidative stress related to impaired glutathione metabolism and mitochondrial dysfunction (177, 178). Both schizophrenia and vascular dementia involve cholinergic dysfunction contributing to cognitive impairment, but schizophrenia is mainly associated with α7 nicotinic acetylcholine receptor (nAChR) abnormalities in frontal–cingulate regions, whereas VaD predominantly affects α4β2 nAChRs in hippocampal and subcortical structures (179).

The evidence suggests a consistently elevated risk of VaD in schizophrenia, though the precise prevalence remains uncertain. The discrepancy likely stems from differences in study populations, diagnostic criteria, and regional healthcare contexts.

## Schizophrenia and fronto-temporal dementia

Evidence suggests a closer relationship between schizophrenia and FTD, particularly behavioural variant FTD (bvFTD), than with AD. Genetic studies support partial etiological overlap: families with FTD show increased rates of schizophrenia, and shared molecular substrates have been identified, including progranulin gene (GRN) mutations, as well as microtubule-associated protein tau (MAPT) and cell adhesion molecule 2 (CADM2) risk genes (180–182). Genome-wide analyses further reveal significant genetic correlations, with dozens of shared genes and pathways implicated in synaptic transmission, immunity, neurodevelopment, and interleukin signaling (183). Rare familial cases describe frontotemporal dementia (FTD) emerging in individuals initially diagnosed with schizophrenia, often linked to GRN, MAPT, or valosin-containing protein (VCP) mutations (184).

Clinically, overlap is substantial. Young-onset FTD may present with prominent psychotic symptoms years before dementia diagnosis, and up to one-third of patients under 30 were initially diagnosed with a primary psychotic disorder (184). Some patients with severe chronic schizophrenia exhibit cognitive deficits resembling bvFTD in both pattern and severity, particularly among inpatients (185). Moreover, in older schizophrenia cohorts, a subset meets formal criteria for dementia—most commonly bvFTD—indicating that neurodegenerative processes may co-occur in vulnerable individuals (186). Despite this clinical and genetic overlap, biomarkers and imaging findings support biological distinctions. Serum neurofilament light chain (NfL) levels are elevated in bvFTD but not in schizophrenia, aiding differential diagnosis (187). MRI studies demonstrate mutation-specific atrophy patterns in FTD, including asymmetric involvement in GRN carriers and more symmetric changes in MAPT mutation cases (188). Together, these findings suggest that schizophrenia and FTD share partial genetic and clinical intersections, yet remain biologically distinguishable entities, with true neurodegeneration emerging in only a subset of patients.

Population-based studies provide the strongest evidence for sex differences in FTD, consistently demonstrating a higher incidence and prevalence in men. An incidence study reported nearly twofold higher rates in men than women (189), and early-onset dementia cohorts similarly showed marked male predominance in FTD (190). These differences appear to vary by subtype and genetic status, with male predominance observed primarily in sporadic behavioral variant FTD but not in genetically determined forms (191). We were unable to identify any studies addressing whether men diagnosed with schizophrenia are at greater risk of developing frontotemporal dementia than women.

**Figure 1:**
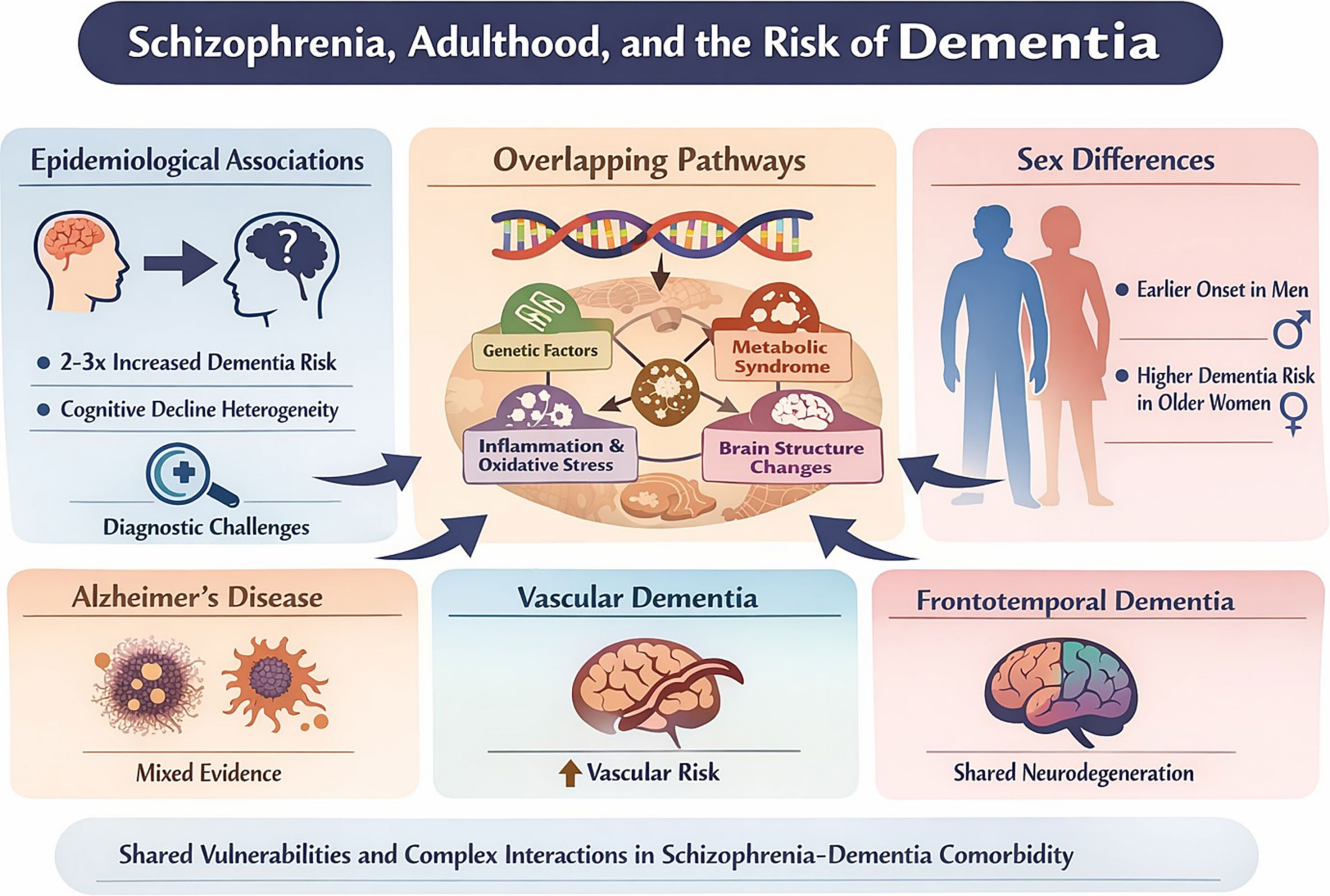
Relationship between schizophrenia and other dementias.

Negative symptoms in schizophrenia—such as apathy, poverty of speech, perseveration, and social withdrawal—closely parallel those observed in patients with frontal lobe injury and FTD, raising the possibility of a shared clinical phenomenon. Both disorders exhibit executive dysfunction, although these deficits tend to be more severe in FTD. The clinical similarity between schizophrenia and FTD may blur diagnostic boundaries, particularly in cases of early-onset FTD or LOS, where overlapping features can lead to misdiagnosis (188). Among 1870 patients, bvFTD-like structural patterns (prefrontal, insular, limbic atrophy) were more common in schizophrenia and major depression than AD-like patterns and were associated with clinical features such as high Body Mass Index, psychomotor slowing, affective disinhibition, and paranoid ideation (192). Meta-analytic evidence indicates a significant spatial overlap in gray matter deficits between first-episode schizophrenia and frontotemporal lobar degeneration, particularly in the bilateral caudate, left insula, and bilateral uncus, suggesting either shared vulnerability of these regions or partially overlapping etiologies (193). Converging neuropsychological, neuroimaging, and neuropathological findings indicate that negative symptoms and cognitive impairments in both disorders are primarily linked to prefrontal cortex dysfunction, while psychotic symptoms such as hallucinations appear more closely associated with temporal lobe pathology (188). Multimodal meta-analyses in bvFTD identify the frontomedian cortex, anterior insula, thalamus, and basal ganglia as core hubs, with partial dissociation between atrophy and hypometabolism. These fronto-insular and subcortical circuits subserve emotion regulation, reward processing, empathy, and executive control, supporting neurobiological overlap between bvFTD and schizophrenia. Deficits in theory of mind may arise secondarily from executive dysfunction, offering a mechanistic explanation for shared clinical features (194). Figure 1 and Table 2 summarize the most important findings from selected studies. Due to word limit constraints, detailed data supporting the FTD column will be provided in the Supplementary materials.

**Table 2:** Key biomarker and neuroimaging profiles in schizophrenia and major dementia syndromes (24, 119, 120, 129, 134, 139, 140, 145–150, 154–158, 160, 187, 188, 194, 222–226).

Biomarker/modality	Schizophrenia	Alzheimer’s disease	Frontotemporal dementia
Amyloid PET	No consistent amyloid accumulation reported	Increased cortical amyloid deposition	Typically negative
CSF Aβ42	Reduced (non-AD pattern; broad Aβ isoform reduction)	Markedly reduced	Normal or mildly reduced
CSF total tau	Normal or reduced	Increased	Normal or mildly reduced
CSF p-tau	Normal or reduced	Increased	Normal
Neurofilament light chain (NfL)	No consistent disease-specific elevation reported	Increased	Increased
APOE ε4 association	No consistent association	Strong risk factor	Studies showing conflicting results
Hippocampal atrophy (MRI)	Present (non-specific)	Progressive and pronounced	Present (less prominent than frontal)
Frontal/orbitofrontal atrophy (MRI)	Present	Mild, typically later-stage	Core feature
White matter changes (MRI/DTI)	Microstructural alterations	Present	Present

CSF, Cerebrospinal Fluid; DTI, Diffusion Tensor Imaging; MRI, Magnetic Resonance Imaging; PET, Positron Emission Tomography.

## Cognitive trajectories after dementia onset in patients with schizophrenia

Data on cognitive trajectories after a formal diagnosis of dementia in individuals with pre-existing schizophrenia remain sparse and inconsistent. While epidemiological studies robustly document an increased *incidence* of dementia in schizophrenia, far fewer investigations address the *rate of cognitive progression* once dementia is established.

In AD, progression in patients with schizophrenia appears to be heterogeneous. Case series and small observational studies suggest both relatively slow cognitive decline despite marked brain atrophy (195, 196) and, conversely, rapid deterioration characterized by impaired learning and accelerated forgetting resembling typical AD trajectories (197). Isolated case reports further document steep short-term declines following dementia onset (198), highlighting substantial interindividual variability. Consistently, a large retrospective cohort study comparing schizophrenia spectrum (*n* = 1,217) and affective disorders (*n* = 2,264) showed that although individuals with schizophrenia experienced earlier cognitive concerns and earlier dementia diagnosis, their rate of cognitive decline was gradual (approximately 1–1.4 MMSE points/year) rather than rapidly progressive, arguing against a predominant fast-decliner phenotype (35). Overall, existing evidence is insufficient to determine whether schizophrenia modifies AD progression in a consistent direction.

Evidence regarding FTD is similarly limited and largely case-based. Several reports describe rapidly progressive courses with marked behavioral and structural deterioration following an initial schizophrenia-like presentation (192, 199, 200), whereas other cases suggest a much slower progression spanning years or even decades, particularly in genetically mediated forms such as chromosome 9 open reading frame 72 (C9orf72)–associated disease (184, 201). Systematic reviews conclude that current data are too fragmentary to reliably characterize progression rates of FTD in patients with schizophrenia (202).

For VaD, we were unable to identify studies directly examining post-diagnostic cognitive trajectories in patients with schizophrenia. Although schizophrenia is associated with accelerated cognitive aging and an increased burden of vascular risk factors (4, 10), subtype-specific data on VaD progression in this population are currently lacking.

Taken together, the available literature suggests an important research gap: while schizophrenia is consistently associated with an elevated risk of dementia, the rate and pattern of cognitive decline following dementia onset remain insufficiently characterized and may vary across dementia subtypes. Furthermore, marked baseline deficits may limit the sensitivity of standard neuropsychological tests to detect subsequent decline due to floor effects, potentially obscuring non-linear cognitive trajectories over the course of illness (2, 203).

## Discussion

Emerging evidence supports the view of schizophrenia as a disorder characterized by both early neurodevelopmental vulnerability and later accelerated or dysregulated aging. Post-mortem studies demonstrate altered expression of synaptic plasticity–related genes, including reduced cyclase-associated protein 2 (CAP2) and discs large MAGUK scaffold protein 1 (DLG1), with CAP2 downregulation also observed in neurodegenerative disorders, suggesting shared mechanisms of synaptic dysfunction (204). Large-scale neuroimaging meta-analyses further show advanced structural brain aging, with a mean brain-predicted age difference of +3.5 years in schizophrenia, independent of clinical characteristics (205). Biological aging markers similarly indicate accelerated aging trajectories associated with increased risk of age-related somatic disease and dementia (206). At the molecular level, age-related gene expression in the prefrontal cortex differs from normal aging, yet partial overlap—particularly in early-stage illness—suggests premature engagement of aging-related pathways (207). Polygenic liability, reflected in higher schizophrenia polygenic risk scores, is associated with poorer cognition, altered hippocampal and fronto-limbic structure and connectivity, and more severe clinical outcomes, suggesting that inherited risk contributes to reduced cognitive reserve already in childhood and adolescence (208, 209). Schizophrenia has been linked to epigenetic alterations, including DNA methylation changes, histone modifications, and dysregulation of non-coding RNAs, which may affect the expression of genes involved in dopaminergic signaling and neurodevelopment (115). Genetic susceptibility likely interacts with prenatal and early-life environmental adversities, disrupting synaptogenesis, myelination, and large-scale network development, which may manifest as lower premorbid IQ and developmental delays (209).

Within a hypothetical integrative framework, schizophrenia can be conceptualized as a condition in which early neurodevelopmental vulnerability interacts with later accelerated aging, increasing susceptibility to cognitive decline and dementia without implying a disorder-specific neurodegenerative pathway (2, 203, 210). Gene-set analyses support this dual framework, indicating contributions from both neurodevelopmental and neurodegeneration-related pathways to brain aging and cognitive decline (211). Both schizophrenia and AD are linked to lower general intelligence, while their direct genetic correlation appears modest but positive, consistent with partially shared yet limited genetic overlap (95, 212). Consequently, some individuals may enter adulthood with reduced cognitive reserve, rendering later-life vascular, metabolic, or age-related insults sufficient to cross clinical thresholds for dementia (24). Based on available evidence, it may be cautiously hypothesized that cognitive resilience—the capacity to maintain cognitive performance despite pathology—is reduced in schizophrenia due to pre-existing cognitive deficits, while resistance, defined as lower-than-expected neuropathological burden, remains unclear and requires biomarker-based investigation (213). Longitudinal neuroimaging studies further suggest that, in a subset of patients, these early vulnerabilities are followed by accelerated or maladaptive aging processes, including early gray matter loss and later progressive white matter deterioration exceeding that expected in normal aging (206, 210, 214). Chronic low-grade neuroinflammation and oxidative stress, observed across the course of schizophrenia, may cumulatively impair neuronal integrity, disrupt the neurovascular unit and blood–brain barrier, and increase vulnerability of white matter and the hippocampus, potentially linking early developmental risk with late cognitive decline (173). Metabolic and vascular comorbidities, including diabetes, hypertension, and metabolic syndrome, may further accelerate cognitive decline by increasing cerebrovascular burden and ischemic injury, thereby elevating the risk of vascular or mixed dementias (215). Taken together, this hypothetical model conceptualizes dementia risk in schizophrenia as an accelerated depletion of cognitive reserve arising from the interaction of early genetic and developmental vulnerability with decades-long exposure to inflammatory, oxidative, lifestyle-related, treatment-related, and somatic factors, rather than as a direct progression to AD (208, 210). Another proposed conceptual framework distinguishes *primary* cognitive impairment, hypothesized to reflect disorder-related neurobiological alterations, from *secondary* cognitive impairment, potentially arising from modifiable factors such as symptoms, pharmacotherapy, metabolic disturbances, or social adversity (216). Within this framework, early cognitive deterioration occurring between the premorbid and prodromal stages—sometimes described as a period of “compressed aging”—may reflect the convergence of primary neurodevelopmental vulnerability with early secondary influences, complicating the interpretation of later-life cognitive trajectories (203). The neurodevelopmental (“static encephalopathy”) model proposes relative stability of cognitive deficits after illness onset, a pattern supported by meta-analytic evidence (2).

Although women with schizophrenia often show better short-term outcomes, menopause-related changes, long-term treatment exposure, and institutionalization have been associated with selective cognitive decline, particularly in processing speed and verbal memory (41, 67, 217). Furthermore, women tend to show greater cognitive resilience in preclinical stages, followed by a disproportionately accelerated cognitive decline and neurodegeneration after the onset of MCI and dementia—resulting in more severe multidomain impairment than men despite comparable amyloid burden (213, 218–221).

**Figure 2:**
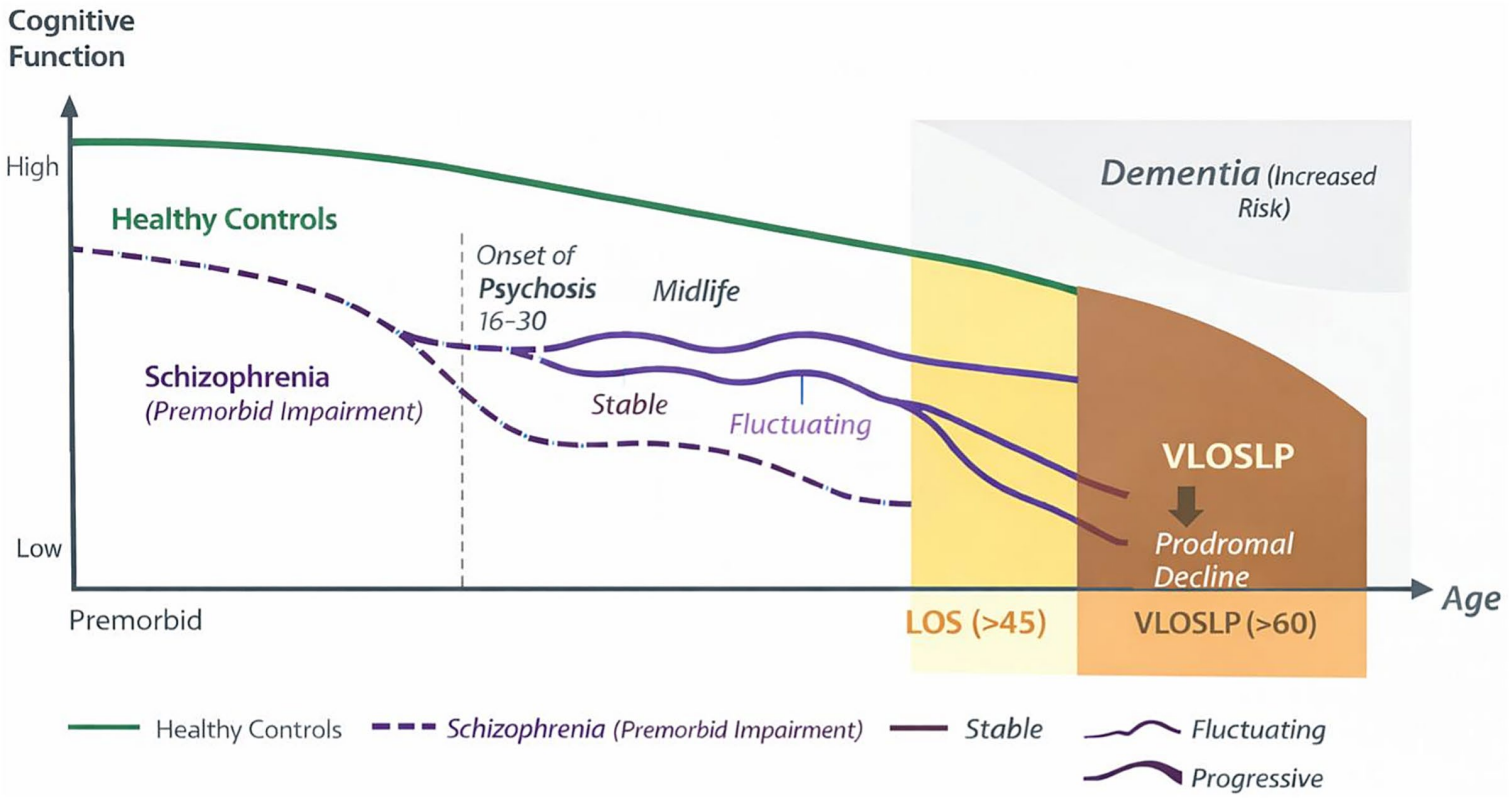
Hypothetical lifespan model of cognitive trajectories in schizophrenia. This schematic illustrates theoretical cognitive trajectories across the lifespan in individuals with schizophrenia compared to healthy controls. The slopes and patterns depicted are conceptual and are not derived from direct longitudinal modeling data. Rather, they represent a synthesis of findings discussed in the present review, including evidence for premorbid impairment, heterogeneous midlife stability or fluctuation, and variable late-life outcomes. The yellow shaded area denotes late-onset schizophrenia (LOS; onset >45 years), while the brown shaded area indicates very-late-onset schizophrenia-like psychosis (VLOSLP; onset >60 years), which has been associated with increased dementia risk and is increasingly conceptualized as a potential prodromal manifestation of neurodegeneration, although causality remains unproven. The shaded dementia field reflects elevated dementia risk reported in epidemiological studies, without implying a uniform or inevitable progression. The figure is intended as an integrative conceptual framework based on the literature reviewed in this article (3, 10, 12, 13, 25, 26, 42, 66, 72, 76, 95, 203, 209).

Current evidence underscores substantial heterogeneity in cognitive profiles and trajectories among individuals with schizophrenia. Accordingly, standardized cognitive assessment should be incorporated into routine clinical practice, using tools sensitive to longitudinal change, accounting for premorbid functioning, and minimizing floor and ceiling effects (46). At the same time, cognitive instruments used in dementia diagnostics may lack specificity in schizophrenia, as positive and negative symptoms, medication effects, and low baseline cognitive reserve can result in dementia-level scores in the absence of primary neurodegenerative pathology (24). Figure 2 shows a hypothetical trajectory of cognitive changes, taking sex differences into account.

## Conclusion

Schizophrenia is linked to persistent cognitive impairment and a markedly elevated risk of dementia, with evidence pointing to heterogeneous cognitive trajectories shaped by both neurodevelopmental vulnerability and later neurodegenerative and cardiometabolic processes. While associations with AD are modest, convergence with frontotemporal is stronger. Overall, schizophrenia confers a multifactorial and clinically diverse risk profile for later-life cognitive decline. Clinically, these findings underscore the need for routine cognitive monitoring, management of cardiometabolic risk factors, and early identification of individuals showing accelerated decline. However, current evidence is limited by diagnostic heterogeneity, short follow-up in many studies, and insufficient integration of biomarker, genetic, and longitudinal cognitive data.

Consideration points:

- Future research could examine whether polygenic risk scores differentiate between individuals with schizophrenia who later develop dementia and those who do not, to disentangle genetic versus environmental contributions.
- Longitudinal studies explicitly examining post-diagnostic cognitive trajectories in patients with schizophrenia and dementia are urgently needed, as current evidence focuses almost exclusively on incidence rather than progression.
- Preventive interventions for individuals at high risk of psychosis, including psychological, pharmacological, and nutritional strategies, have shown modest benefits in meta-analyses, though further well-controlled trials are needed. Emerging evidence highlights the potential of compounds targeting inflammation and oxidative stress, such as omega-3 fatty acids and N-acetyl cysteine, while biomarker-based risk stratification remains a key future priority (3).
