# Supplementary material for: Schizophrenia and dementia across the lifespan: epidemiological links, cognitive trajectories, and the pathophysiological interplay
Source: Front Neurol. 2026 Apr 7;17:1779076. doi: 10.3389/fneur.2026.1779076 (PMC13096775; doi:10.3389/fneur.2026.1779076)
Supplement: Supplementary file 1 [file Data_Sheet_1.PDF]

Literature Search Strategy (Supplementary Materials)

This narrative review was based on a structured literature search conducted primarily in PubMed. The search term "schizophrenia AND dementia" was applied, covering publications from 1994 to 2025. The initial search yielded 1,419 records. Titles and abstracts were screened for relevance to the topic of dementia risk, cognitive decline, and neurodegenerative processes in schizophrenia. After preliminary screening, 150 articles were retained for further evaluation. Following full-text assessment, 101 articles were selected for detailed analysis and inclusion in the review.

Preference was given to longitudinal cohort studies, registry-based studies, meta-analyses, and high-quality review articles addressing long-term cognitive trajectories and incident dementia. Studies were excluded if they:

- included very small sample sizes,
- were not available in English,
- did not directly address dementia risk or cognitive decline in schizophrenia,
- or focused exclusively on unrelated psychiatric or neurological conditions.

During the peer-review process, additional relevant publications were incorporated to address specific reviewer comments (e.g., sex differences, hormonal influences, metabolic factors, and menopause-related mechanisms in schizophrenia and Alzheimer's disease). Some of these studies were not captured by the initial keyword-based PubMed search due to their focus on specific biological modifiers rather than explicitly indexed dementia outcomes. These articles were identified through targeted supplementary searches and reference tracking to ensure conceptual completeness of the review.

We acknowledge that a substantial number of publications remained outside the final selection. This reflects the broad and heterogeneous nature of the topic, encompassing schizophrenia, late-onset schizophrenia (LOS), very-late-onset schizophrenia-like psychosis (VLOSLP), Alzheimer's disease, vascular dementia, frontotemporal dementia, and related biological mechanisms. The final selection aimed to balance comprehensiveness with relevance and thematic coherence.

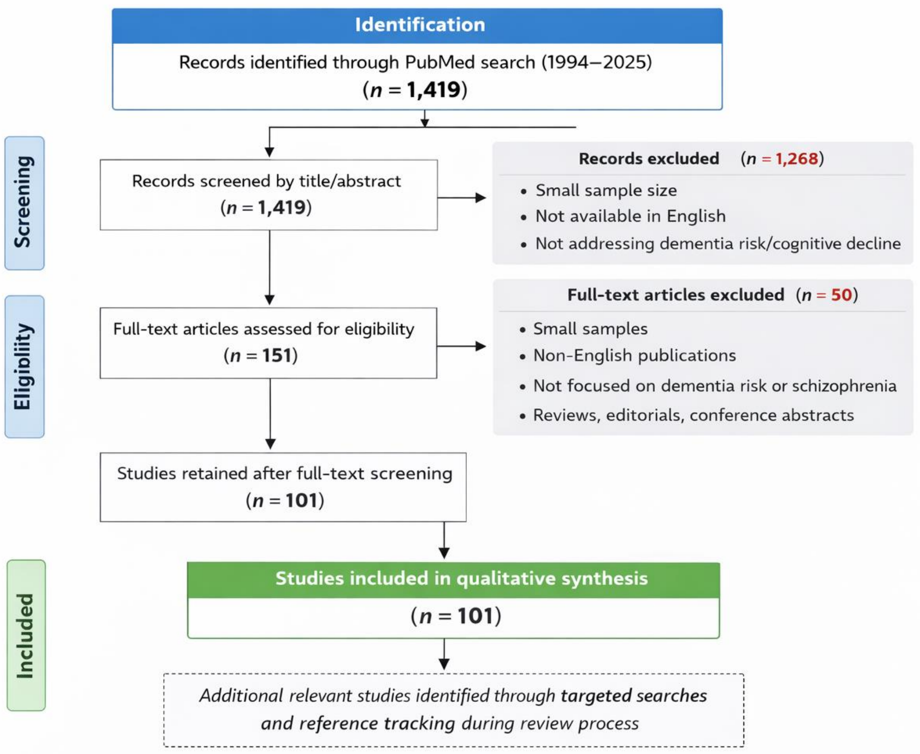

Cerebrospinal fluid (CSF) biomarker profiles in frontotemporal dementia (FTD) show distinct patterns compared to Alzheimer's disease (AD). CSF tau levels are significantly reduced in many FTD patients, with 34% showing significantly low tau levels, which is never observed in AD (Grossman et al., 2005). In behavioral variant FTD, CSF tau levels negatively correlate with visual association performance, while Aβ42 levels positively correlate with general cognitive function and executive abilities (Koedam et al., 2013). Approximately 21% of FTD patients exhibit an AD-like CSF pattern (high tau, low Aβ42), which correlates with hypoperfusion in brain regions typically affected by AD (Padovani et al., 2013). In familial FTD with tau mutations (P301L and G272V), CSF total tau levels are only mildly increased compared to controls, while phosphorylated tau-181 levels remain normal, contrasting with the elevated levels seen in AD (Rosso et al., 2003). In FTD, CSF tau levels are elevated compared with controls but lower than in AD, whereas Aβ42 is reduced relative to controls but higher than in AD; the greatest diagnostic accuracy in differentiating FTD from AD is achieved using tau/Aβ42 ratios, particularly p-tau/Aβ42 (Riemenschneider et al., 2002; Casoli et al., 2019). These findings demonstrate CSF biomarkers' utility in differentiating FTD from AD.

The relationship between APOE ε4 and frontotemporal dementia (FTD) remains controversial, with studies showing conflicting results. In a Chinese population study, Ji et al. (2013) found significantly increased APOE ε4 allele frequency in FTD patients (16.13%) compared to controls (7.34%), suggesting it may be a risk factor for both FTD and AD. However, Geschwind et al. (1998) reported contrasting findings in their study of 33 rigorously diagnosed FTD patients, where APOE ε4 frequency was 21% - significantly lower than in early-onset (38%) and late-onset (40%) patients with AD, and not significantly different from elderly controls (13%). Interestingly, Mehta et al. (2007) identified APOE ε4 as a potential modifier gene specifically in inclusion-body myopathy, Paget's disease, and frontotemporal dementia (IBMPFD), an autosomal dominant form of FTD, where APOE ε4 genotype was significantly associated with FTD development ($P = 0.0002$).

References

Casoli, T., Paolini, S., Fabbietti, P., Fattoretti, P., Paciaroni, L., Fabi, K., Gobbi, B., Galeazzi, R., Rossi, R., Lattanzio, F., & Pelliccioni, G. (2019). Cerebrospinal fluid biomarkers and cognitive status in differential diagnosis of frontotemporal dementia and Alzheimer's disease. *The Journal of international medical research*, *47*(10), 4968–4980. https://doi.org/10.1177/0300060519860951

Geschwind, D., Karrim, J., Nelson, S. F., & Miller, B. (1998). The apolipoprotein E epsilon4 allele is not a significant risk factor for frontotemporal dementia. *Annals of neurology*, *44*(1), 134–138. https://doi.org/10.1002/ana.410440122

Grossman, M., Farmer, J., Leight, S., Work, M., Moore, P., Van Deerlin, V., Pratico, D., Clark, C. M., Coslett, H. B., Chatterjee, A., Gee, J., Trojanowski, J. Q., & Lee, V. M. (2005). Cerebrospinal fluid profile in frontotemporal dementia and Alzheimer's disease. *Annals of neurology*, *57*(5), 721–729. https://doi.org/10.1002/ana.20477

Ji, Y., Liu, M., Huo, Y. R., Liu, S., Shi, Z., Liu, S., Wisniewski, T., & Wang, J. (2013). Apolipoprotein E ε4 frequency is increased among Chinese patients with frontotemporal dementia and Alzheimer's disease. *Dementia and geriatric cognitive disorders*, *36*(3-4), 163–170. https://doi.org/10.1159/000350872

Koedam, E. L., van der Vlies, A. E., van der Flier, W. M., Verwey, N. A., Koene, T., Scheltens, P., Blankenstein, M. A., & Pijnenburg, Y. A. (2013). Cognitive correlates of cerebrospinal fluid biomarkers in frontotemporal dementia. *Alzheimer's & dementia : the journal of the Alzheimer's Association*, *9*(3), 269–275. https://doi.org/10.1016/j.jalz.2011.12.007

Mehta, S. G., Watts, G. D., Adamson, J. L., Hutton, M., Umberger, G., Xiong, S., Ramdeen, S., Lovell, M. A., Kimonis, V. E., & Smith, C. D. (2007). APOE is a potential modifier gene in an autosomal dominant form of frontotemporal dementia (IBMPFD). *Genetics in medicine : official journal of the American College of Medical Genetics*, *9*(1), 9–13. https://doi.org/10.1097/gim.0b013e31802d830d

Padovani, A., Premi, E., Pilotto, A., Gazzina, S., Cosseddu, M., Archetti, S., Cancelli, V., Paghera, B., & Borroni, B. (2013). Overlap between frontotemporal dementia and Alzheimer's disease: cerebrospinal fluid pattern and neuroimaging study. *Journal of Alzheimer's disease : JAD*, *36*(1), 49–55. https://doi.org/10.3233/JAD-121969

Riemenschneider, M., Wagenpfeil, S., Diehl, J., Lautenschlager, N., Theml, T., Heldmann, B., Drzezga, A., Jahn, T., Förstl, H., & Kurz, A. (2002). Tau and Abeta42 protein in CSF of patients with frontotemporal degeneration. *Neurology*, *58*(11), 1622–1628. https://doi.org/10.1212/wnl.58.11.1622

Rosso, S. M., van Herpen, E., Pijnenburg, Y. A., Schoonenboom, N. S., Scheltens, P., Heutink, P., & van Swieten, J. C. (2003). Total tau and phosphorylated tau 181 levels in the cerebrospinal fluid of patients with frontotemporal dementia due to P301L and G272V tau mutations. *Archives of neurology*, *60*(9), 1209–1213. https://doi.org/10.1001/archneur.60.9.1209
